# Adult and children’s use of hand sanitizer during a pandemic – an observational study

**DOI:** 10.1038/s41370-022-00479-w

**Published:** 2022-09-24

**Authors:** Theresa K. Lopez, Kelly Jones, Ann Roseberry-Lincoln, Angelika Zidek, Leona MacKinnon, Leonora Marro

**Affiliations:** 1 Tetra Tech, 10711 Red Run Blvd., Suite 105, Owings Mills, MD 21117 USA; 2 Tetra Tech, Regina, SK Canada; 3 Tetra Tech, Owings Mills, MD USA; 4 Healthy Environments and Consumer Safety Branch, Health Canada, Ottawa, ON Canada

**Keywords:** Hand sanitizer, Exposure, Consumer, School, Children

## Abstract

**Background:**

The use of hand sanitizers has been one of the key public health measures recommended to reduce the transmission of SARS-CoV-2 during the pandemic. As such, its daily use among the general population has reportedly increased dramatically since the onset of the COVID-19 pandemic.

**Objective:**

To better understand the impact of this recommendation, hand sanitizer use, including the frequency and amount handled, was examined among adults in a non-occupational setting and children in both the home and school/childcare settings.

**Methods:**

An online survey of Canadians (conducted from September to October 2021) was employed to estimate use frequency, amount, and pattern of hand sanitizer use.

**Results:**

Responses were received from 655 adults in the general population and 298 teachers of children up to the age of 18 years. The frequency of hand sanitizer use during the pandemic was found to be as high as 25 times per day in children and over 9 times per day in adults. Notable differences were found when comparing the frequency of hand sanitizer use by children in the home to children in a school or childcare setting.

**Significance:**

This is the first study, known to the authors, examining hand sanitizer use among children during the pandemic, including use in a childcare or school setting. This study illustrates the importance of examining the change in consumer behaviors during a pandemic and the need to look beyond the home when attempting to understand product use patterns in children.

**Impact statement:**

This research explores uses of hand sanitizer, before and during pandemic conditions, in the general population of Canada with a particular focus on use among children. The results can be used to estimate exposure to chemicals in hand sanitizer from non-occupational use in Canada and among similar populations and signal the importance of examining changing consumer behaviors and use of consumer products in school settings, especially among children.

## Introduction

Since the start of the COVID-19 pandemic, public health officials have stressed the importance of proper hygiene, with increased advice on using hand sanitizers in schools and childcare settings [1]. To maintain good hand hygiene when soap and water are not available, use of a hand sanitizer containing at least 60% alcohol is recommended by public health organizations. In addition to active ingredients, hand sanitizers may also contain other substances () and impurities. Exposures to substances and impurities in hand sanitizers are assessed as part of a chemical risk assessment using key exposure parameters such as frequency of use and amount used. The most common active ingredients in hand sanitizers include ethyl alcohol, 2-propanol and benzalkonium chloride [2], representing ~96% of the reported active ingredients in hand sanitizers [3]. Many of these substances may cause skin burns, eye damage or irritation, dizziness and/or may cause cancer [4–6]. A number of regulators, including Health Canada, the Dutch National Institute for Public Health and the Environment (RIVM) and the European Commission Scientific Committee on Consumer Safety (SCCS) have examined the safety of chemicals found in hand sanitizers [7–9]. More recently, the Dutch government looked specifically at use of ethanol-containing hand sanitizers by consumers and workers during the pandemic, including examining use at up to 25 times/day in children and up to 100 times/day in adolescents and adults [8]. The COVID-19 global pandemic has altered the use pattern of hand sanitizers and highlighted the need for more relevant information that may signal changing consumer behaviors and increased exposures.

Chemical exposure is infrequently measured or reported in the literature. Therefore, parameters that affect the level of exposure are identified and modeling is used to estimate consumer exposure. Such parameters include frequency of use by age group, route of exposure (such as dermal, ingestion, or inhalation), amount used, duration of product use, and location of use. Some sources of information, including the scientific literature [10–13] and ConsExpo fact sheets, compile exposure information for groups of products including cleaning products, cosmetics, do-it-yourself products, pest control, and children’s toys [14]. However, consumer use of hand sanitizer has changed in response to the COVID-19 pandemic, and pre-pandemic research is unlikely to reflect current use patterns. Further, previously conducted research of hand sanitizer use did not specifically include use in a school/childcare setting [10].

Statistics Canada reported a 792% increase in the sales of hand sanitizer in March 2020 compared to the same period in 2019, and businesses noted a continued high demand for hand sanitizer through 2021 [15, 16]. No other literature quantifying use of hand sanitizer in the Canadian general population has been identified, although there are reports of increased eye injury in children from unintentional contact of hand sanitizer with the eye [17, 18]. The Canadian Surveillance System for Poison Information also noted up to a 400% increase in hand sanitizer related poison center calls between January and June 2020 compared to previous years [19].

Some Canadian public health messaging encouraged children to clean their hands “after going to the washroom, before eating, after coughing, sneezing or blowing their nose, after playing with shared toys, after touching animals and after outdoor activities” [1]. Based on this advice, children may be applying hand sanitizers in schools and childcare settings 10–15 times/day. However, there is limited information on the frequency of use of hand sanitizers by children in schools or childcare settings. Children in particular are considered a vulnerable population with respect to chemical exposures, as their exposures would be higher than adults due to their lower body weights and increased hand-to-mouth behaviors [20]. Given the advice to increase hand hygiene, the possibility of overexposure to chemicals in hand sanitizers and potential underestimation of chemical exposure in risk assessment was identified as an area for further investigation.

## Objective

The objective of this study was to collect and synthesize Canadian-specific information regarding non-occupational use of hand sanitizer products in adults and children. Increased use of hand sanitizer during the COVID-19 pandemic created the need to reexamine use patterns to ensure human health risk assessments are protective of actual exposures, particularly for children.

## Methods

The current study collected hand sanitizer use information in support of human health risk assessments under Canada’s Chemical Management Plan. Hand sanitizers in the form of gel, liquid or foam dispensed by pump, squeeze, or spray were the focus of the survey. To collect information, this study used two online surveys (available in English and French): a general population survey and a survey aimed at teachers and childcare providers.

### Study population

Three target Canadian populations were identified for the survey: adults (≥18 years old); children (<18 years old) in the home (specifically non-school/childcare setting); and children in a school or childcare setting. The first target population was defined as adults across all geographic regions of Canada who personally use hand sanitizer in a non-work setting (general population study); this population was also asked if individuals <18 years old were living in their home. Adult respondents were asked about their personal use of hand sanitizer as well as use of hand sanitizer by children in the home, if applicable. A second survey of teachers and childcare providers was conducted to ascertain the use of hand sanitizer in school or childcare settings by children <18 years. In both surveys, the time period of interest was defined as “during pandemic,” from March 2020 (when internationally a pandemic had been declared) to the current day (data collection ended on October 9, 2021). “Prior to the pandemic” was defined as before March 2020, while “after the pandemic” was undefined in terms of an exact date. The general population survey also inquired whether respondents used hand sanitizer before the pandemic and whether their use after the pandemic would be more, less, or equally as frequent.

In the general population study, adult respondents were asked if children <18 years of age resided in their home and if so, to identify the ages of those children (grouped as <2 years of age, 2 years, and 3 years, and then 2-year age groups for example 4–5 years, 6–7 years, etc.). If the adult chose more than two age groups living in the home, they were asked to evaluate no more than 2 randomly selected age groups from their responses. Similarly, teachers/childcare providers were asked to specify which age groups they supervised and were asked about a maximum of 2 randomly selected age groups.

### Survey design and data collection

The Dynata/Research Now Canada consumer panel was used to provide a sample source to achieve a representative number of online survey participants in the target populations. Dynata/Research Now recruits and maintains consumer panels in markets worldwide including members contacted via a variety of online sites and forums. At the time of data collection, the Dynata/Research Now Canada panel included approximately one million English and French language Canadians, representing a cross-section of Canadian residents. Respondents receive a small compensation for their participation, equivalent to ~$2.00CAD.

A random sample of Canadian adults in all provinces and territories was selected to receive the online survey. To qualify for the survey, adults must have used hand sanitizer in the past 6 months. For the survey of teacher/childcare providers, they must identify as a teacher/childcare provider at the time of the survey. Data collection was conducted from September 23, 2021, to October 9, 2021. A total response of 655 adults from the general population survey was obtained; 326 teachers responded to the teacher survey in total.

Survey questions were developed for gel, liquid, or foam hand sanitizer formulations dispensed as a pump, squeeze, or spray. The survey allowed respondents to select more than one type of hand sanitizer that they used or observed being used. In the event that multiple types of hand sanitizer were selected, questions were posed for up to 2 types of hand sanitizer, prioritizing dispenser types least often selected.

Age and demographic information were collected for the general population. The first question in the general population survey established that the respondent had used hand sanitizer in the past 6 months; to prevent bias, the question also asked respondents if they had used hand cream/lotion, sunscreen lotion, or body cream/lotion. If the respondent did not report hand sanitizer use, the online survey was terminated.

The teacher/childcare provider survey questions were designed to first collect demographic and occupation information about the adult participant, followed by questions about their observation of a group of minors using hand sanitizer in a school or childcare setting. The teacher/childcare provider survey collected responses that represent “in-school” use patterns for children aged 4–18 years.

For both surveys, the observational questions addressed age of participant and/or minor(s), product type, use location (for example, bathroom, car, or in the classroom), use frequency, and amount used. Daily use frequency options were described in several categories and differed between the general population and teacher/childcare surveys. Typical amount of product usage was described in terms of number of pumps (for liquid, foam, and gel), squeezes (for liquid and gels) or sprays (for spray forms only). If questions in each survey allowed for responses as “other,” the specific response was reviewed for appropriate classification.

### Data analyses

Data were tabulated in Microsoft Excel 365 while data summaries, descriptive analyses, and comparative visualizations were performed using R (v4.0.2) [21–23]. All response data for both surveys were categorical variables. The number of responses and percentages (95% confidence intervals) per age group or overall were tabulated for frequencies and amounts of hand sanitizer used; 95% confidence intervals were calculated assuming a binomial distribution. Data were evaluated separately and visualized for general population and the teacher/childcare surveys. Similarly, data for adults were evaluated separately from data for children. Adult data were reviewed for patterns by age group. All data regarding children were separated according to age groups for comparison of in-home and at-school use patterns. Chi-squared tests performed to determine difference in frequency or amount used between age groups showed no statistically significant difference due to small sample size. The Cochran–Mantel–Haenszel (CMH) test (performed using SAS [24]) was used to test for significant differences between home and school frequency of use and amount of product used in those aged 4–17 years, after adjusting for age. The general association *p*-value of the CMH test has been reported here.

In the general population survey, six adult respondents checked that they used liquid spray. However, in the question about amount of spray used during the pandemic, each of the six selected “zero sprays” as the option. These six results were removed prior to analyzing the amount of spray used by adults during the pandemic; their responses were retained for all other forms of hand sanitizer.

## Results

### Observations for general population

Participants consisted of 655 individuals (318 males, 335 females, 2 other) from the 10 provinces of Canada. No survey responses were received from the 3 territories. The age of respondents ranged from 19–85 years, with an arithmetic mean of 44.3 years. Most respondents were from Ontario (*n* = 311); followed by Alberta (*n* = 82), Quebec (*n* = 70), and British Columbia (*n* = 63).

#### Adults

**Fig. 1:**
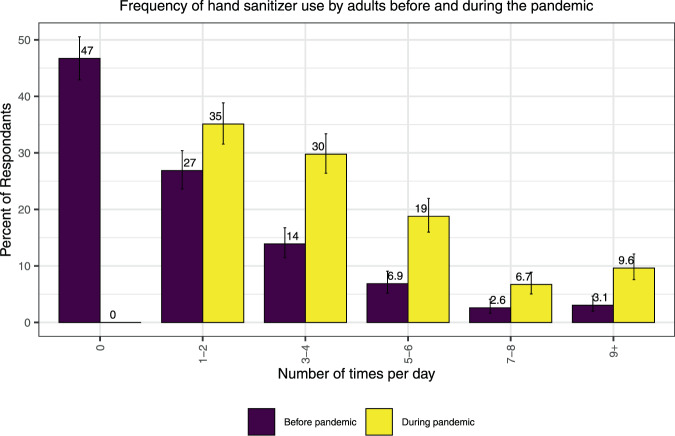
Frequency of hand sanitizer use by adults before and during the pandemic. The frequency of use as self-reported by adult respondents (%) before the pandemic presented in the dark bar; during the pandemic shown in the light bar, with 95% confidence interval range shown as a line.

Most adults (*n* = 468, 71%) reported more frequent use of hand sanitizer during the pandemic than before it. Some reported the same frequency of use (*n* = 162, 25%) and very few adults reported a decrease in use (*n* = 25, 3.8%). Nearly half of the adults (47%) responded that they did not use hand sanitizer (0 times/day) before the pandemic (Fig. 1). Further, more than half of adults (55%) indicated their use of hand sanitizers would remain the same once the pandemic was over while 31% would use it less. Overall, 65% of adults reported using hand sanitizer in a non-work setting up to 4 times/day while 35% of respondents reported use of 5 or more applications per day (Table 1), with 16% of respondents reporting use of 7 or more applications per day. In contrast, prior to the pandemic, 41% used it up to 4 times/day while ~13% used it 5 or more times/day (Fig. 1). No significant differences were seen between males and females.

**Table 1:** Frequency of hand sanitizer use by adults in different age groups^a^.

Times per day	19–34 *n* = 244	35–49 *n* = 168	50–64 *n* = 168	65+ *n* = 75	Total *n* = 655
**1–2**	32 (27–38) *n* = 79	29 (23–36) *n* = 49	39 (32–46) *n* = 65	49 (38–60) *n* = 37	35 (32–39) *n* = 230
**3–4**	30 (25–36) *n* = 73	31 (24–38) *n* = 52	27 (21–34) *n* = 45	33 (24–45) *n* = 25	30 (26–33) *n* = 195
**5–6**	21 (17–27) *n* = 52	20 (15–27) *n* = 34	15 (11–22) *n* = 26	15 (8.4–24) *n* = 11	19 (16–22) *n* = 123
**7–8**	7.8 (5–12) *n* = 19	7.1 (4.1-12) *n* = 12	6.5 (3.7–11) *n* = 11	2.7 (0.73–9.2) *n* = 2	6.7 (5–8.9) *n* = 44
**9**+	8.6 (5.7–13) *n* = 21	12 (8.3–18) *n* = 21	12 (8.3–18) *n* = 21	*n* = 0	9.6 (7.6–12) *n* = 63

^a^Values represent percent of adults in each age group or overall with the 95% confidence interval in parentheses; *n* equals number of adults in the category.

The pump form of hand sanitizer (gel, liquid, or foam) appears to be most often used (*n* = 578, 88% of all responses), and gel pump (*n* = 437, 41%) more often than liquid pump (*n* = 382, 36%) or foam pump (*n* = 254, 24%). Respondents reported using squeeze forms less often than pump (squeeze gel, *n* = 287; squeeze liquid, *n* = 213) and spray form the least often (*n* = 214). Adults most frequently reported using hand sanitizer during a pandemic in public buildings (*n* = 573) followed by the car (*n* = 441), bathroom (*n* = 262), and kitchen (*n* = 210). Use in other places in the home (*n* = 160) and outside (*n* = 105) were also reported. These categorical responses were not exclusive; respondents could select all that applied.

The amount of hand sanitizer used during the pandemic was most often reported as 1 pump, squeeze, or spray (Table 2). Two or more pumps, squeezes or sprays were reported by 22%, 25% and 47% of adults, respectively. Three or more pumps or squeezes was reported by <5% of adults, and 15% of adults for sprays.

**Table 2:** Amount of hand sanitizer used by adults in different age groups^a^.

Amount	19–34	35–49	50–64	65 +	Total	
Pump	*n* = 146	*n* = 121	*n* = 121	*n* = 55	*n* = 443	
0.5	21 (15–28) *n* = 30	12 (7.7–19) *n* = 15	14 (9–21) *n* = 17	27 (17-40) *n* = 15	17 (14–21) *n* = 77	
1	54 (46–62) *n* = 79	69 (61–77) *n* = 84	61 (52–69) *n* = 74	55 (42–67) *n* = 30	60 (56–65) *n* = 267	
2	21 (15–29) *n* = 31	14 (9–21) *n* = 17	21 (15–30) *n* = 26	18 (10–30) *n* = 10	19 (16–23) *n* = 84	
3+	4.1 (1.9–8.7) *n* = 6	4.1 (1.8–9.3) *n* = 5	3.3 (1.3–8.2) *n* = 4	*n* = 0	3.4 (2.1–5.5) *n* = 15	
Squeeze	*n* = 158	*n* = 95	*n* = 86	*n* = 33	*n* = 372	
0.5	16 (11–22) *n* = 25	17 (11–26) *n* = 16	17 (11–27) *n* = 15	21 (11–38) *n* = 7	17 (13–21) *n* = 63	
1	58 (50–66) *n* = 92	57 (47–66) *n* = 54	56 (45–66) *n* = 48	67 (50–80) *n* = 22	58 (53–63) *n* = 216	
2	23 (17–30) *n* = 36	20 (13–29) *n* = 19	21 (14–31) n = 18	12 (4.8–27) *n* = 4	21 (17–25) *n* = 77	
3+	3.2 (1.4–7.2) *n* = 5	6.3 (2.9–13) *n* = 6	5.8 (2.5–13) *n* = 5	*n* = 0	4.3 (2.7–6.9) *n* = 16	
Spray	*n* = 89	*n* = 49	*n* = 48	*n* = 22	*n* = 208	
0.5	6.7 (3.1–14) *n* = 6	8.2 (3.2–19) *n* = 4	8.3 (3.3–20) *n* = 4	*n* = 0	6.7 (4.1–11) *n* = 14	
1	39 (30–50) *n* = 35	47 (34–61) *n* = 23	40 (27–54) *n* = 19	82 (61–93) *n* = 18	46 (39–52) *n* = 95	
2	36 (27–46) *n* = 32	24 (15–38) *n* = 12	40 (27–54) *n* = 19	18 (7.3–39) *n* = 4	32 (26–39) *n* = 67	
3+	18 (11–27) *n* = 16	20 (11–34) *n* = 10	12 (5.9–25) *n* = 6	*n* = 0	15 (11–21) *n* = 32	

Respondents provided amounts for up to two types each.

^a^Values represent percent of adults in each age group and type with the 95% confidence interval in parentheses; n equals number of adults in the category.

#### Children

Of the respondents from the general population survey, 231 of the 655 individuals (35%) reported having children in the home and completed the survey regarding use of hand sanitizer by those children. All age groups (<2–17 years old) had at least one response, for a total of 310 children in the different age groups. Responses were secured from 9 of the 10 provinces; no responses for children were received from Prince Edward Island. More than one form of dispenser could be selected, but “pump” (*n* = 259, 84%) was chosen most often and “spray” (*n* = 34, 11%) was least often selected. Gel (*n* = 205, 66 %) was the most frequently selected form of hand sanitizer overall for all age groups.

Adults reported that children in the home used hand sanitizer more during the pandemic than prior and most respondents stated that use of hand sanitizer by children will remain the same or increase after the pandemic. Adults reported more frequently assisting children aged ≤7 years with application of hand sanitizer, with 77% of those providing assistance reporting helping this age range in some way.

**Fig. 2:**
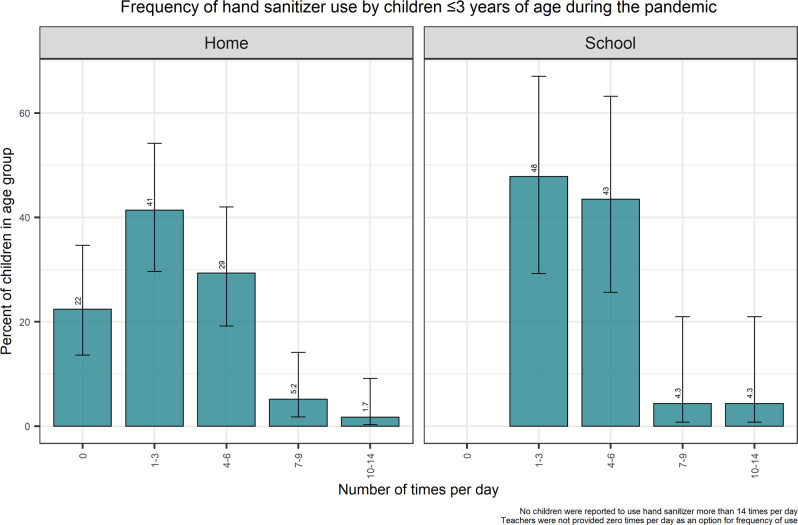
Frequency of hand sanitizer use in children ≤ 3 years of age during the pandemic. Frequency of hand sanitizer use in children ≤ 3 years of age as reported by adult caretakers at home (left) or at school (right), with 95% confidence interval range shown as a line.

It was reported that 0 or 1–3 applications of hand sanitizer per day was most frequent among children aged <2 years in a home setting during the pandemic, while the majority of 2-year-olds were reported to use hand sanitizer 4–6 times/day (Fig. 2, Supplementary Table 1). There were no reports of 15–25 uses of hand sanitizer per day in the ≤3-year-old group. In children aged 4–17 years, 35% were reported to use hand sanitizer 1–3 times/day and 58% were reported to use 4 or more times/day (Table 3). Application of hand sanitizer 15–25 times per day when at home was reported in 3.2% of respondents overall (Table 3) and was not reported in age groups 4–5 or 8–9 years.

**Table 3:** Frequency of hand sanitizer use by children aged ≥4 years at home or at school as reported by the adult respondent^a^.

Times per day	4–5 years	6–7 years	8–9 years	10–11 years	12–13 years	14–15 years	16–17 years	Total
Home	*n* = 45	*n* = 32	*n* = 34	*n* = 49	*n* = 37	*n* = 23	*n* = 32	*n* = 252
0	13 (6.3–26) *n* = 6	*n* = 0	12 (4.7–27) *n* = 4	6.1 (2.1–17) *n* = 3	*n* = 0	4.3 (0.77–21) *n* = 1	9.4 (3.2–24) *n* = 3	6.7 (4.3–11) *n* = 17
1–3	38 (25–52) *n* = 17	38 (23–55) *n* = 12	41 (26–58) *n* = 14	29 (18–42) *n* = 14	35 (22–51) *n* = 13	35 (19–55) *n* = 8	34 (20–52) *n* = 11	35 (30–41) *n* = 89
4–6	20 (11–34) *n* = 9	31 (18–49) *n* = 10	26 (15–43) *n* = 9	31 (20–45) *n* = 15	43 (29–59) *n* = 16	30 (16–51) *n* = 7	34 (20–52) *n* = 11	31 (25–36) *n* = 77
7–9	24 (14–39) *n* = 11	19 (8.9–35) *n* = 6	18 (8.3–34) *n* = 6	18 (10–31) *n* = 9	11 (4.3–25) *n* = 4	22 (9.7–42) *n* = 5	12 (5–28) *n* = 4	18 (14–23) *n* = 45
10–14	4.4 (1.2–15) *n* = 2	6.2 (1.7–20) *n* = 2	2.9 (0.52–15) *n* = 1	14 (7.1–27) *n* = 7	2.7 (0.48–14) *n* = 1	4.3 (0.77–21) *n* = 1	6.2 (1.7–20) *n* = 2	6.3 (3.9–10) *n* = 16
15–25	*n* = 0	6.2 (1.7–20) *n* = 2	*n* = 0	2 (0.36–11) *n* = 1	8.1 (2.8–21) *n* = 3	4.3 (0.77–21) *n* = 1	3.1 (0.55–16) *n* = 1	3.2 (1.6–6.1) *n* = 8
School	*n* = 72	*n* = 98	*n* = 75	*n* = 18	*n* = 34	*n* = 65	*n* = 62	n = 424
1–3	29 (20–41) *n* = 21	15 (9.5–24) *n* = 15	13 (7.4–23) *n* = 10	33 (16–56) *n* = 6	2.9 (0.52–15) *n* = 1	29 (20–41) *n* = 19	24 (15–36) *n* = 15	21 (17–25) *n* = 87
4–6	42 (31–53) *n* = 30	51 (41–61) *n* = 50	48 (37–59) *n* = 36	22 (9–45) *n* = 4	53 (37–69) *n* = 18	42 (30–54) *n* = 27	44 (32–56) *n* = 27	45 (41–50) *n* = 192
7–9	17 (9.8–27) *n* = 12	15 (9.5–24) *n* = 15	21 (14–32) *n* = 16	28 (12–51) *n* = 5	21 (10–37) *n* = 7	14 (7.5–24) *n* = 9	16 (9–27) *n* = 10	17 (14–21) *n* = 74
10–14	6.9 (3–15) *n* = 5	13 (7.9–21) *n* = 13	13 (7.4–23) *n* = 10	5.6 (0.99–26) *n* = 1	18 (8.3–34) *n* = 6	11 (5.3–21) *n* = 7	11 (5.6–22) *n* = 7	12 (8.9–15) *n* = 49
15–25	5.6 (2.2–13) *n* = 4	5.1 (2.2–11) *n* = 5	4 (1.4–11) *n* = 3	11 (3.1–33) *n* = 2	5.9 (1.6–19) *n* = 2	4.6 (1.6–13) *n* = 3	4.8 (1.7–13) *n* = 3	5.2 (3.5–7.7) *n* = 22

Frequency of hand sanitizer use by children aged 4 years and older at home or at school as reported by the adult respondent. Zero was not provided as an option for the teacher survey.

^a^Values represent percent of children in each age group and location with the 95% confidence interval in parentheses; *n* equals number of children in the category.

**Fig. 3:**
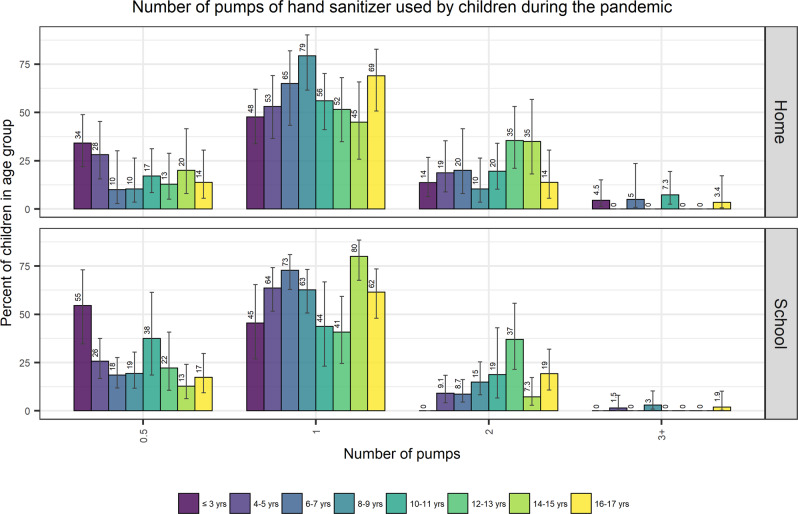
Amount of pump form of hand sanitizer used by children in home and school settings as reported by adult respondents. Amount of pump form of hand sanitizer used per application by children (as reported by adult caretakers), by increasing age group from left to right, with 95% confidence interval range shown as line.

Amounts of hand sanitizer per use among children ≤3 years old at home was most often reported as 1 pump or 0.5 squeezes (Fig. 3, Supplementary Table 2). Spray hand sanitizer was the least often selected option for type of hand sanitizer in the home, with only 6 reports of children aged ≤3 years using this form (Supplementary Table 2). In children aged 4–17 years, use of a pump form of hand sanitizer was most often reported (Fig. 3), followed by the squeeze form with only 28 reports of use of spray (Supplementary Table 3).

### Observations for teachers and childcare responses

Three hundred twenty-six responses were collected from the independent teacher/childcare-oriented survey. Responses were collected from all 10 provinces, with the most responses from Ontario (*n* = 157), Alberta (*n* = 41), and British Columbia (*n* = 36). No responses were received from the 3 territories. Approximately 95% of respondents reported teaching or supervising children aged <18 years, while the rest taught adults or in a setting unrelated to children. Only responses from teachers/childcare providers whose students were <18 years were included in the analysis (*n* = 298 teachers, representing 7 age grouped responses for children). Respondents could provide answers for up to two different age groups, if they supervised more than one age group. In these responses, a “day” reflects a school day.

The respondent could select all answers that applied for type of hand sanitizer and location of use, while frequency and amount per use selections were exclusive categories. Pump form of dispenser (*n* = 375, 76.1%) was most often reported as being used in school/childcare settings by children aged 4–17 years, with gel pump (*n* = 235) being the most frequently reported. Spray (*n* = 66, 13.4%) was the second most common form of dispensing reported and squeeze forms were least frequently used (*n* = 52, 10.5%). Location of use in schools/childcare facilities was most often the classroom/playroom for all age groups (*n* = 385), followed by the hallway (*n* = 215). Bathroom (*n* = 190) and lunchroom (*n* = 201) were less frequent locations of use, and outside was least frequent (*n* = 114).

Teachers and childcare providers were asked how often hand sanitizer is used by or applied to the children that they supervise. This response for frequency of use at school may not capture the full extent of use in a given day by a child (e.g., application at home before or after school). It was most frequently reported (45%) that hand sanitizers were used 4–6 times/day across all age groups (4–17 years old) in school/childcare settings with about 34% reporting a use of between 7–25 times/day and 21% reporting 1–3 times/day (Table 3). Approximately 17% of 4–17 year olds in the school/childcare survey reported a frequency of use ranging from 10–25 times/day. In the youngest age group (≤3 years old), 91% used hand sanitizer up to 6 times per day and <5% reported use of 7–9 or 10–14 times/day (Fig. 2). There were no reports of use >14 times per day in the ≤3 years age group.

The amount of hand sanitizer used per application was most frequently answered as one pump, squeeze, or spray (Supplementary Table 3). When a pump dispenser was reported, 85% reported that one or half a pump was dispensed per application in children aged 4–17 years in a school setting; only 1% reported using ≥3 pumps per application. One squeeze (60%) and one spray (67%) were also the most frequently reported in this group followed by 2 squeezes (27%) or 2 sprays (20%). Few reported ≥3 squeezes (4%) or sprays (7%). There were no reports of ≥3 pumps, squeezes or sprays in the ≤3-year-old age group in school/childcare settings (Supplementary Table 2). Overall, there were more reports of use of spray in a school setting (*n* = 67) than a home setting (*n* = 34). Therefore, it seems that while spray is least often used, the number of sprays per application tends to be higher than pump or squeeze forms (Supplementary Table 3).

Frequency and amount used reported by adults for children at home and school/daycare were compared for 4–17-year-olds. There was a statistically significant difference in the frequency of use between home and school (*p* < 0.0001), in particular children between 4–17 years old were more likely to use hand sanitizer 4–6 times/day while at school, whereas most of the children at home reported using hand sanitizer 1–3 times/day (Table 3). There was no statistically significant difference in the amount of pump or squeeze product used (*p* > 0.05 in both cases) in schools and homes. Regarding the amount of spray product used, there was a marginal statistically significant difference between the amount of product used at home compared to at school, after adjusting for age (*p* = 0.04); home children were more likely to use >2 sprays while school children were more likely to use 1 spray.

## Discussion

This is the first study to examine Canadian-specific exposure associated with the reported use of hand sanitizer by the general population and children in a school/childcare setting; and the first study assessing hand sanitizer use among children during a pandemic (to the knowledge of the authors). This information is expected to support regulators in estimating exposures when assessing human health risk from chemicals in hand sanitizers.

The entire study was conducted by engaging participants in an online survey, rather than using other methods such as interviews, phone calls, or mailings. While these surveys were offered to all Canadian provinces and territories, no responses were received from the territories. The combination of a small number of panelists in the territories, the necessity of having used hand sanitizer in the past six months or self-identifying as a teacher contributed to this non-response. Income level was not asked of respondents. To the extent that income level may affect hand sanitizer use, it is unknown what impact this may have on the reported range of uses in the general population responses. This is unlikely to affect the teacher survey.

Consumer exposure is addressed in a variety of different ways, especially when evaluating consumer behavior information. Wu et al. 2010 examined the frequency of use of waterless hand sanitizers via telephone interviews (with structured questionnaires) of 604 California (USA) households and found that between 41–65% of adult respondents used waterless hand sanitizers with mean frequencies of use for adults ranging from ~0.4–3 uses/day (P90 ~1.4–8 uses/day), and for children ranging from ~0.7–1.4 uses/day (P90 ~2–5 uses/day) [10]. RIVM more recently published an assessment of the use of ethanol in hand sanitizers for workers and consumers during the pandemic; given the lack of published use data, assumed a range of frequencies of 1–25/day for 0–11-year-olds and 1–100/day for >11-year-olds [8]. Alsaidan et al. 2020 conducted a self-administered online questionnaire of students and employees of a Saudi Arabian university that included questions on use of hand sanitizer during the COVID-19 pandemic [25]. Of the 2356 respondents, 87.6% reported that their habit of using hand sanitizers had changed during the pandemic, with 20.9% of respondents using it more than 10 times per day. The results from this Canadian study show slightly lower frequencies of use during the pandemic in adults in a non-work setting (90.4% reporting 1–8 uses/day, and 9.6% reporting >9 uses/day) when compared to Alsaidan et al. 2020 [25] but indicates slightly higher frequencies of use compared to results from Wu et al. 2010 [10] during non-pandemic times. Nearly half of the adults (47%) responded that they did not use hand sanitizer before the pandemic (Fig. 1) which is similar to the percentage of adult non-users (35–59%) reported in Wu et al. 2010 [10] during non-pandemic times.

For children in the home, 82% reported use of hand sanitizer during the pandemic between 1 and 9 times/day, with 9.7% reporting no use in the home and 8.1% reporting >10 uses/day. However, in school/childcare settings, it was most frequently reported (45%) that hand sanitizers were used 4–6 times/day across 4–17-year-olds in school/childcare settings with 34% reporting a use of between 7–25 times/day. This is also the first study to provide evidence of use of hand sanitizers in Canadian children <3 years of age. Use of hand sanitizers in the home and in childcare settings for this age group had frequencies ranging from 1 to as high as 14 times/day during the pandemic. In contrast, Wu et al. 2010 reported (for non-pandemic circumstances) mean frequencies of use for 0–1-year-olds of ~0.65 uses/day with a P90 of 2.5 uses/day and ~0.78 uses/day for children 2–5 years with a P90 of 2 uses/day. In particular, this study highlights the importance of surveying teachers and childcare providers on child-specific behaviors. Use of certain products by children in the classroom or in childcare facilities may be higher than in a home environment.

Product amounts reported to be used by children ≤3 years ranged from 0.5 to >3 pumps, squeezes, or sprays. In the home, pump products were often reported as 1 pump whereas squeeze products were more often reported as 0.5 squeeze; most reported >3 sprays for this age group. In school/ childcare settings for ≤3 years, 0.5 and 1 pump, squeeze or spray were most often reported. The volume of hand sanitizer dispensed from the various types of products was not investigated in this survey. Based on available data, the volume of product dispensed from pump forms of hand sanitizer can vary greatly with the volume from 1 pump of product ranging from 0.4 to 1.75 mL [26–31]. Data on the amount of product released from spray forms was not identified.

Frequency of reported use was statistically greater in schools (4–6 times/day) compared to home settings (1–3 times/day). Children most often used the pump form of hand sanitizer in both home and school settings, and the amount dispensed per use was similar at home compared to at school. This may reflect that product dispensers at schools (automatic or dispensed by an adult) dispense a controlled amount.

More frequent use at school may be due to the recommendations for frequent hand sanitizing when in public spaces, as well as less access to a sink for hand washing when in a school/childcare setting. Because there was no recommended or mandated use of hand sanitizer in school/childcare settings prior to the pandemic, all use of hand sanitizer in a school setting was viewed as an increase. In a pre-pandemic survey, the 90^th^ percentile frequency of use reported in children aged 5 and older was 3 per day for females and 5 per day for males, although these observations were not in a school setting [10]. The findings of the current survey indicated that reported use of hand sanitizer of 10–25 times/day occurs in all age groups in a school/ childcare setting including 24% of 12–13-year-olds and 18% of 6–7-year-olds. For children at home, almost all age groups reported 10–25 uses/day with the highest frequency of 16% for children 10–11 years old.

A comparison of exposure by children using the pre-pandemic frequency of use reported in Wu et al. 2010 [10] to school-aged children in this study was conducted. Exposures were conservatively estimated assuming: one pump of hand sanitizer per use (~1.5 mL/pump and equivalent to 1.5 g/use for simplicity); complete absorption via dermal or inhalation; a body weight of 18.6 kg [13]; using a mean frequency of use of 0.8 times/day to represent pre-pandemic exposure [10]; and using a frequency of use of 25 times/day to represent pandemic exposure for a child in school. The comparison showed an increase in exposure of about 10–30 times (Supplementary Table 4).

Most adults reported an increase in use of hand sanitizer during the pandemic; less than 4% reported a decrease in use. Most adults reported that their use would increase (14%) or remain the same as it was during the pandemic (55%), while 31% reported that it would decrease after the pandemic. For children in the home, parents reported that most (44%) would continue to use the same amount on their child, and that 29% would use more hand sanitizer after the pandemic. Again, this predicted increase in use may relate to increase in time spent in public spaces but may also be related to children returning to school or other activities that will increase both their contact with others and increased independence.

The survey gathered responses from adults who observed their own child/children as well as teachers/childcare providers reporting on the use of hand sanitizer by the children that they supervise. Some uncertainties exist when using parent or teacher observations, and when grouping and comparing responses from the surveys. For example, a slight difference in age groups was used between the two surveys, and it is expected that a person responding for a group would respond with an average amount that had been observed. A parent or caretaker reporting on product use by their own children may be more specific than a teacher/childcare provider in a classroom setting with multiple children to supervise. It should be noted that results from this survey do not represent the entire daily use pattern in adults or children as it did not include workplace use of hand sanitizers for adults or consider use in multiple settings for children (e.g., at home, school/childcare, after-school activities, etc.).

## Conclusions

Overall, daily use of hand sanitizer during a pandemic was higher when compared to use before the COVID-19 pandemic for adults at home, with a typical use frequency of up to 6 times per day and reports of >9 times/day during the pandemic. More importantly, most adults indicated that their use of hand sanitizers would remain the same post-pandemic. During the pandemic, the reported use of hand sanitizer among children at home or in a school or childcare setting was as many as 21–25 times per day. In children, the frequency of use of hand sanitizer is higher in schools or childcare settings than in the home but the amount per use was similar.

This study also provides evidence of use of hand sanitizers in children ≤3 years and the importance of including this vulnerable subpopulation when estimating exposures and potential risks to substances in these products. Finally, data compiled in this study supports a potential shift in consumer behaviors post-pandemic as responses confirmed an increase in use during the pandemic for both adults and children, which may inform risk assessments on substances used in hand sanitizers.

## Significance

The responses gathered from the surveys help to fill gaps related to hand sanitizer use and actual behavior of Canadian adults and children representing all age groups. The responses are expected to provide improved exposure parameters and descriptions related to location and amounts used, and inform regulators on changing consumer behaviors during and after a pandemic. These values may help inform exposure estimates for human health risk assessments and potential changing consumer use patterns related to public health emergencies. This study also highlights the importance of examining the use of products by children in a school or childcare setting where product use may be higher than in the home.

## Supplementary information

Reporting Checklist

Supplementary Information

Supplementary Information

## Data Availability

The questionnaires that were used for data collection are available as supplementary material to this publication. Additional data are available from the corresponding author on reasonable request.
