# Supplementary material for: Adult and children’s use of hand sanitizer during a pandemic – an observational study
Source: J Expo Sci Environ Epidemiol. 2022 Sep 24;33(6):1004–12. doi: 10.1038/s41370-022-00479-w (PMC9510540; doi:10.1038/s41370-022-00479-w)
Supplement: Supplementary file 2 — Supplementary Information [file 41370_2022_479_MOESM2_ESM.pdf]

Supplementary Information

This supplement contains four supplementary tables with more detailed use information for children's age groups in both home and school settings, as well as an exposure assessment based on pre-pandemic and during-pandemic hand sanitizer use among children. In addition, it contains the survey questions used in collecting data for the general population survey and teacher/childcare survey regarding hand sanitizer use.

Supplemental Table 1. Frequency of hand sanitizer use by children aged ≤3 years at home or at school as reported by the adult respondent. [(1)]

	Home				School
**Times per day**	**<2 yrs n=18**	**2 yrs n=18**	**3 yrs n=22**	**Total n=58**	**≤3 yrs n=23**
**0**	39 (20-61) n=7	17 (5.8-39) n=3	14 (4.7-33) n=3	22 (14-35) n=13	*
**1-3**	39 (20-61) n=7	22 (9-45) n=4	59 (39-77) n=13	41 (30-54) n=24	48 (29-67) n=11
**4-6**	17 (5.8-39) n=3	44 (25-66) n=8	27 (13-48) n=6	29 (19-42) n=17	43 (26-63) n=10
**7-9**	n=0	17 (5.8-39) n=3	n=0	5.2 (1.8-14) n=3	4.3 (0.77-21) n=1
**10-14**	5.6 (0.99-26) n=1	n=0	n=0	1.7 (0.31-9.1) n=1	4.3 (0.77-21) n=1
**15-25**	n=0	n=0	n=0	n=0	n=0

* Not provided as an option

(1) Values represent percent of children in each age group and location with the 95% confidence interval in parentheses; n equals number of children in the category.

Supplemental Table 2. Amount of hand sanitizer used by children aged ≤3 years at home or at school by product type as reported by the adult respondent. [(1)]

	Home				School
**Amount**	**<2 yrs**	**2 yrs**	**3 yrs**	**Total**	**≤3 yrs**
**Pump**	n=14	n=13	n=17	n=44	n=22
**0.5**	36 (16-61)<br>n=5	38 (18-64)<br>n=5	29 (13-53)<br>n=5	34 (22-49)<br>n=15	55 (35-73)<br>n=12
**1**	43 (21-67)<br>n=6	46 (23-71)<br>n=6	53 (31-74)<br>n=9	48 (34-62)<br>n=21	45 (27-65)<br>n=10
**2**	14 (4.0-40)<br>n=2	7.7 (1.4-33)<br>n=1	18 (6.2-41)<br>n=3	14 (6.4-27)<br>n=6	n=0
**3+**	7.1 (1.3-31)<br>n=1	7.7 (1.4-33)<br>n=1	n=0	4.5 (1.3-15)<br>n=2	n=0
**Squeeze**	n=6	n=8	n=7	n=21	n=1
**0.5**	50 (19-81)<br>n=3	38 (14-69)<br>n=3	57 (25-84)<br>n=4	48 (28-68)<br>n=10	100 (21-100)<br>n=1
**1**	33 (9.7-70)<br>n=2	25 (7.1-59)<br>n=2	43 (16-75)<br>n=3	33 (17-55)<br>n=7	n=0
**2**	17 (3.0-56)<br>n=1	12 (2.2-47)<br>n=1	n=0	9.5 (2.7-29)<br>n=2	n=0
**3+**	n=0	25 (7.1-59)<br>n=2	n=0	9.5 (2.7-29)<br>n=2	n=0
**Spray**	n=2	n=3	n=1	n=6	n=1
**0.5**	n=0	33 (6.1-79)<br>n=1	n=0	17 (3.0-56)<br>n=1	n=0
**1**	50 (9.5-91)<br>n=1	n=0	100 (21-100)<br>n=1	33 (9.7-70)<br>n=2	100 (21-100)<br>n=1
**2**	n=0	n=0	n=0	n=0	n=0
**3+**	50 (9.5-91)<br>n=1	67 (21-94)<br>n=2	n=0	50 (19-81)<br>n=3	n=0

(1) Values represent percent of children in each age group and location with the 95% confidence interval in parentheses; n equals number of children in the category. Respondents provided amounts for up to two types per child.

Supplemental Table 3. Amount of hand sanitizer used by children aged ≥4 years at home or at school by product type as reported by the adult respondent. [(1)]

	4-5 yrs		6-7 yrs		8-9 yrs		10-11 yrs		12-13 yrs		14-15 yrs		16-17 yrs		Total	
**Amount**	**Home**	**School**	**Home**	**School**	**Home**	**School**	**Home**	**School**	**Home**	**School**	**Home**	**School**	**Home**	**School**	**Home**	**School**
**Pump**	n=32	n=66	n=20	n=92	n=29	n=67	n=41	n=16	n=31	n=27	n=20	n=55	n=29	n=52	n=202	n=375
**0.5**	28 (16-45) n=9	26 (17-37) n=17	10 (2.8-30) n=2	18 (12-28) n=17	10 (3.6-26) n=3	19 (12-30) n=13	17 (8.5-31) n=7	38 (18-61) n=6	13 (5.1-29) n=4	22 (11-41) n=6	20 (8.1-42) n=4	13 (6.3-24) n=7	14 (5.5-31) n=4	17 (9.4-30) n=9	16 (12-22) n=33	20 (16-24) n=75
**1**	53 (36-69) n=17	64 (52-74) n=42	65 (43-82) n=13	73 (63-81) n=67	79 (62-90) n=23	63 (51-73) n=42	56 (41-70) n=23	44 (23-67) n=7	52 (35-68) n=16	41 (25-59) n=11	45 (26-66) n=9	80 (68-88) n=44	69 (51-83) n=20	62 (48-74) n=32	60 (53-66) n=121	65 (60-70) n=245
**2**	19 (8.9-35) n=6	9.1 (4.2-18) n=6	20 (8.1-42) n=4	8.7 (4.5-16) n=8	10 (3.6-26) n=3	15 (8.3-25) n=10	20 (10-34) n=8	19 (6.6-43) n=3	35 (21-53) n=11	37 (22-56) n=10	35 (18-57) n=7	7.3 (2.9-17) n=4	14 (5.5-31) n=4	19 (11-32) n=10	21 (16-27) n=43	14 (10-17) n=51
**3+**	n=0	1.5 (0.27-8.1) n=1	5 (0.89-24) n=1	n=0	n=0	3 (0.82-10) n=2	7.3 (2.5-19) n=3	n=0	n=0	n=0	n=0	n=0	3.4 (0.61-17) n=1	1.9 (0.34-10) n=1	2.5 (1.1-5.7) n=5	1.1 (0.42-2.7) n=4
**Squeeze**	n=20	n=8	n=17	n=8	n=13	n=7	n=21	n=1	n=16	n=6	n=10	n=11	n=9	n=11	n=106	n=52
**0.5**	25 (11-47) n=5	25 (7.1-59) n=2	29 (13-53) n=5	n=0	7.7 (1.4-33) n=1	14 (2.6-51) n=1	29 (14-50) n=6	n=0	12 (3.5-36) n=2	n=0	50 (24-76) n=5	9.1 (1.6-38) n=1	11 (2-43) n=1	9.1 (1.6-38) n=1	24 (17-33) n=25	9.6 (4.2-21) n=5
**1**	65 (43-82) n=13	50 (22-78) n=4	47 (26-69) n=8	75 (41-93) n=6	69 (42-87) n=9	57 (25-84) n=4	57 (37-76) n=12	n=0	25 (10-49) n=4	50 (19-81) n=3	40 (17-69) n=4	82 (52-95) n=9	56 (27-81) n=5	45 (21-72) n=5	52 (42-61) n=55	60 (46-72) n=31
**2**	5 (0.89-24) n=1	25 (7.1-59) n=2	18 (6.2-41) n=3	25 (7.1-59) n=2	23 (8.2-50) n=3	29 (8.2-64) n=2	9.5 (2.7-29) n=2	100 (21-100) n=1	44 (23-67) n=7	50 (19-81) n=3	10 (1.8-40) n=1	n=0	22 (6.3-55) n=2	36 (15-65) n=4	18 (12-26) n=19	27 (17-40) n=14
**3+**	5 (0.89-24) n=1	n=0	5.9 (1-27) n=1	n=0	n=0	n=0	4.8 (0.85-23) n=1	n=0	19 (6.6-43) n=3	n=0	n=0	9.1 (1.6-38) n=1	11 (2-43) n=1	9.1 (1.6-38) n=1	6.6 (3.2-13) n=7	3.8 (1.1-13) n=2
**Spray**	n=4	n=9	n=8	n=13	n=3	n=13	n=4	n=3	n=3	n=6	n=2	n=12	n=4	n=10	n=28	n=66
**0.5**	25 (4.6-70) n=1	11 (2-43) n=1	n=0	n=0	n=0	7.7 (1.4-33) n=1	25 (4.6-70) n=1	n=0	n=0	n=0	n=0	8.3 (1.5-35) n=1	n=0	10 (1.8-40) n=1	7.1 (2-23) n=2	6.1 (2.4-15) n=4
**1**	n=0	56 (27-81) n=5	12 (2.2-47) n=1	69 (42-87) n=9	100 (44-100) n=3	69 (42-87) n=9	25 (4.6-70) n=1	100 (44-100) n=3	33 (6.1-79) n=1	83 (44-97) n=5	100 (34-100) n=2	58 (32-81) n=7	50 (15-85) n=2	60 (31-83) n=6	36 (21-54) n=10	67 (55-77) n=44
**2**	50 (15-85) n=2	22 (6.3-55) n=2	12 (2.2-47) n=1	15 (4.3-42) n=2	n=0	15 (4.3-42) n=2	25 (4.6-70) n=1	n=0	33 (6.1-79) n=1	17 (3-56) n=1	n=0	33 (14-61) n=4	50 (15-85) n=2	20 (5.7-51) n=2	25 (13-43) n=7	20 (12-31) n=13
**3+**	25 (4.6-70) n=1	11 (2-43) n=1	75 (41-93) n=6	15 (4.3-42) n=2	n=0	7.7 (1.4-33) n=1	25 (4.6-70) n=1	n=0	33 (6.1-79) n=1	n=0	n=0	n=0	n=0	10 (1.8-40) n=1	32 (18-51) n=9	7.6 (3.3-17) n=5

(1) Values represent percent of children in each age group and location with the 95% confidence interval in parentheses; n equals number of children in the category. Respondents provided amounts for up to two types of hand sanitizer per child.

Supplemental Table 4. **Estimated systemic exposures (mg/kg-bw/day) pre- and during pandemic to unspecified chemical present in hand sanitizer at various concentrations and used by children aged 5 years. (1)**

Concentration	Pre-Pandemic(2)	Pandemic(3)
**1%**	0.65	20.16
**15%**	9.68	302.42
**60%**	32.26	1008.06

(1) Systemic exposure estimated = frequency of use (/day) x concentration of substance in hand sanitizer (fraction) x amount of product used (1500 mg/use) / body weight (18.6 kg)

(2) Frequency of use of 0.8 times per day (mean value for 2-5 year olds reported in Wu et al. 2010)

(3) Frequency of use of 25 times per day (high end value reported in this study)

General Population Survey

This survey targeted adults across all geographic regions of Canada who personally use hand sanitizer in a non-work setting. Participants were asked about their personal use of hand sanitizer. If they had children <18 years of age living at home, they were asked about hand sanitizer use for up to two children. The survey contained the questions and possible responses shown below. For each categorical question, unchecked responses were stored as 0 and checked responses were stored as 1.

Questions about the Adult

Preliminary 1. Please provide the first three digits of your postal code. [These digits provided the province where the respondent resided.]

1. Newfoundland
2. Nova Scotia
3. Prince Edward Island
4. New Brunswick
5. Quebec
6. Ontario
7. Manitoba
8. Saskatchewan
9. Alberta
10. British Columbia
11. Atlantic (Newfoundland, Nova Scotia, Prince Edward Island, New Brunswick)
12. Prairies (Manitoba, Saskatchewan, Alberta)
13. West (Manitoba, Saskatchewan, Alberta, British Columbia)

Preliminary 2. What is your gender?

1. Male
2. Female
3. Other

Preliminary 3. What is your age? [Respondent filled in an integer value which was placed in one of the following categories.]

1. 19 – 34 years
2. 35 – 49 years
3. 50 – 64 years
4. 65+ years

Question 1. Which of the following products have you used in the past six months? [Multiple responses possible]

1. Body cream or lotion
2. Hand cream or lotion
3. Hand sanitizer
4. Sunscreen lotion
5. None of the above

Question 2. What type of hand sanitizer product(s) have you used, or do you use? [Multiple responses possible]

1. Foam pump
2. Gel pump
3. Liquid pump
4. Gel squeeze
5. Liquid squeeze
6. Liquid spray
7. None of the above

The survey selected up to two types (pump, squeeze, or spray), biasing the selection toward underrepresented types, to obtain details about use.

Question 3. During the pandemic, when thinking about foam, gel, or liquid pump hand sanitizers, how much product do you typically use with each application? [One response only]

1. ½ pump
2. 1 pump
3. 2 pumps
4. 3 or more pumps

Question 4. Prior to the pandemic, when thinking about foam, gel, or liquid pump hand sanitizer, how much product did you typically use with each application? [One response only]

1. Did not use
2. ½ pump
3. 1 pump
4. 2 pumps
5. 3 or more pumps

Question 5. During the pandemic, when thinking about gel or liquid squeeze hand sanitizers, how much product do you typically use with each application? [One response only]

1. ½ squeeze
2. 1 squeeze
3. 2 squeezes
4. 3 or more squeezes

Question 6. Prior to the pandemic, when thinking about gel or liquid squeeze hand sanitizers, how much product did you typically use with each application? [One response only]

1. Did not use
2. ½ squeeze
3. 1 squeeze
4. 2 squeezes
5. 3 or more squeezes

Question 7. During the pandemic, when thinking about spray forms of hand sanitizer, how much product do you typically use with each application? [One response only]

1. ½ spray
2. 1 spray
3. 2 sprays
4. 3 or more sprays

Question 8. Prior to the pandemic, when thinking about spray forms of hand sanitizer, how much product did you typically use with each application? [One response only]

1. Did not use
2. ½ spray
3. 1 spray
4. 2 sprays
5. 3 or more sprays

Question 9. During the pandemic, on a typical day (not at work), how many times per day do you use hand sanitizer? [One response only]

1. 1 – 2
2. 3 – 4
3. 5 – 6
4. 7 – 8
5. 9 or more

Question 10. During the pandemic, where do you use hand sanitizer, on a typical day (not at work)? [Multiple responses possible]

1. In the car
2. In stores or other public places
3. In the kitchen
4. In the bathroom
5. Other places in the home
6. Outdoors

Question 11. Prior to the pandemic, on a typical day (not at work), how many times per day do you use hand sanitizer? [One response only]

1. Did not use
2. 1 – 2
3. 3 – 4
4. 5 – 6
5. 7 – 8
6. 9 or more

Question 12. Once the pandemic is over, will you likely use hand sanitizers less, the same, or more than you have during the pandemic? [One response only]

1. Less
2. Same
3. More

Question 13a. Do you have a child or children aged 0 – 18 years in your household currently? [One response only]

1. Yes
2. No

Question 13b. How old is your child (or each child in the case of multiple children)? [Multiple responses possible].

1. <2 years
2. 2 years
3. 3 years
4. 4 – 5 years
5. 6 – 7 years
6. 8 – 9 years
7. 10 – 11 years
8. 12 – 13 years
9. 14 – 15 years
10. 16 – 17 years

Questions about Children at Home

The following questions were asked for up to two children in the household, with the results tied to the age of the children selected by the survey.

Question 14. During the pandemic, how often do you apply hand sanitizer to your child or does your child use hand sanitizer (excluding use at a school or childcare setting)? [One response only]

1. 1 – 3/day
2. 4 – 6/day
3. 7 – 9/day
4. 10 – 14/day
5. 15 – 20/day
6. 21 – 25/day
7. More than 25 times per day (please specify how many)
8. Did not use

Question 15. Prior to the pandemic, how often did you apply hand sanitizer to your child or did your child use hand sanitizer (excluding use at a school or childcare setting)? [One response only]

1. Did not use
2. 1 – 3/day
3. 4 – 6/day
4. 7 – 9/day
5. 10 – 14/day
6. 15 – 20/day
7. More than 25 times per day (please specify how many)

Question 16. During the pandemic what type of product is typically applied to/used by your child? [Multiple responses possible]

1. Foam pump
2. Gel pump
3. Liquid pump
4. Gel squeeze
5. Liquid squeeze
6. Liquid spray

The survey selected up to two types(pump, squeeze, or spray), biasing the selection toward underrepresented types, to obtain details about use.

Question 17. During the pandemic when thinking about foam, gel, or liquid pump hand sanitizer, how much product is typically applied to or used by your child with each application? [One response only]

1. ½ pump
2. 1 pump
3. 2 pumps
4. 3 or more pumps

Question 18. During the pandemic when thinking about gel or liquid squeeze hand sanitizer, how much product is typically applied to or used by your child with each application? [One response only]

1. ½ squeeze
2. 1 squeeze
3. 2 squeezes
4. 3 or more squeezes

Question 19. During the pandemic when thinking about spray forms hand sanitizer, how much product is typically applied to or used by your child with each application? [One response only]

1. ½ spray
2. 1 spray
3. 2 sprays
4. 3 or more sprays

Question 20. Who applied the product (child or adult)? [One response only]

1. Child self-applies product or product is auto-dispensed
2. Adult applies/assists

Question 21. How was assistance provided to the child? [One response only]

1. Product dispensed into child's hand by adult
2. Child applies product but with some assistance (such as help with rubbing product on hands)
3. Product dispensed into adult's hand and then rubbed into child's hand by adult
4. Other. Please specify.

Question 22. After the pandemic is over, how frequently do you think you will apply hand sanitizer to your child or that your child will use hand sanitizer? [One response only]

1. Same as during the pandemic
2. More than during the pandemic
3. Less than during the pandemic, but more than prior to the pandemic
4. Less than during the pandemic, and less than or similar to use prior to the pandemic
5. Will not continue to use hand sanitizer
6. Unsure

Teacher/Childcare Provider Survey

This survey targeted teachers and childcare providers across all geographic regions of Canada who personally supervise children in a school or daycare setting. Participants were asked about hand sanitizer use for up to two children. The survey contained the questions and possible responses shown below. For each categorical question, unchecked responses were stored as 0 and checked responses were stored as 1.

Questions about the Teacher/Childcare Provider

Preliminary 1. Please provide the first three digits of your postal code. [These digits provided the province where the respondent resided.]

1. Newfoundland
2. Nova Scotia
3. Prince Edward Island
4. New Brunswick
5. Quebec
6. Ontario
7. Manitoba
8. Saskatchewan
9. Alberta
10. British Columbia
11. Atlantic (Newfoundland, Nova Scotia, Prince Edward Island, New Brunswick)
12. Prairies (Manitoba, Saskatchewan, Alberta)
13. West (Manitoba, Saskatchewan, Alberta, British Columbia)

Preliminary 2. What is your gender?

1. Male
2. Female
3. Other

Preliminary 3. What is your age? [Respondent filled in an integer value which was placed in one of the following categories.]

1. 19 – 34 years
2. 35 – 49 years
3. 50 – 64 years
4. 65+ years

Preliminary 4. Which of the following best describes the industry segment of the company/organization for which you work? [One response only]

1. Business services
2. Consulting
3. Childcare services
4. Education
5. Government
6. Not-for-profit
7. Other

Preliminary 5. At what education/childcare provider level are you employed? [One response only]

1. Preschool
2. Elementary school/primary school
3. Middle school/junior high
4. High school/senior high
5. District K-12
6. College/university
7. Other. Please specify.

Question 1. What age group(s) of children do you typically supervise? [Multiple responses possible]

1. ≤3 years
2. 4 – 5 years
3. 6 – 7 years
4. 8 – 9 years
5. 10 – 11 years
6. 12 – 13 years
7. 14 – 15 years
8. 16 – 17 years

Questions about Children at School/Daycare

The following questions were asked for up to two children in the household, with the results tied to the age of the children selected by the survey.

Question 2. During the pandemic when thinking about the [selected age group] that you supervise, what type of hand sanitizer do they use while under your supervision? [Multiple responses possible]

1. Foam pump
2. Gel pump
3. Liquid pump
4. Gel squeeze
5. Liquid squeeze
6. Liquid spray
7. Do not use

The survey selected up to two types(pump, squeeze, or spray), biasing the selection toward underrepresented types, to obtain details about use.

Question 3. During the pandemic when thinking about foam, gel, or liquid pump hand sanitizer, how much product is typically applied to or used by your child with each application? [One response only]

1. ½ pump
2. 1 pump
3. 2 pumps
4. 3 or more pumps

Question 4. During the pandemic when thinking about gel or liquid squeeze hand sanitizer, how much product is typically applied to or used by your child with each application? [One response only]

1. ½ squeeze
2. 1 squeeze
3. 2 squeezes
4. 3 or more squeezes

Question 5. During the pandemic when thinking about spray forms hand sanitizer, how much product is typically applied to or used by your child with each application? [One response only]

1. ½ spray
2. 1 spray
3. 2 sprays
4. 3 or more sprays

Question 6. During the pandemic, how often is hand sanitizer used by or applied to the children that you supervise? [One response only]

1. 1 – 3/day
2. 4 – 6/day
3. 7 – 9/day
4. 10 – 14/day
5. 15 – 20/day
6. 21 – 25/day
7. More than 25 times per day (please specify how many)

Question 7. During the pandemic, where has the application of hand sanitizer typically taken place? [Multiple responses possible]

1. Bathroom
2. Lunchroom/kitchen
3. Outdoors
4. Classroom/playroom
5. Hallway

Question 8. Who applied the product (child or childcare provider/teacher)?

1. Child self-applies product or product is auto-dispensed
2. Childcare provider or teacher applies/assists

Question 9. How was assistance provided to the child? [One response only]

1. Product dispensed into child's hand by adult
2. Child applies product but with some assistance (such as help with rubbing product on hands)
3. Product dispensed into adult's hand and then rubbed into child's hand by adult
4. Other. Please specify.
