# Supplementary material for: Adult and children’s use of hand sanitizer during a pandemic – an observational study
Source: J Expo Sci Environ Epidemiol. 2022 Sep 24;33(6):1004–12. doi: 10.1038/s41370-022-00479-w (PMC9510540; doi:10.1038/s41370-022-00479-w)
Supplement: Supplementary file 3 — Supplementary Information [file 41370_2022_479_MOESM3_ESM.docx]

**Supplementary Information**

Adult Survey Questions

Teacher Survey Questions

Supplemental Table 1. Frequency of hand sanitizer use by children aged ≤3 years at home or at school as reported by the adult respondent.

Supplemental Table 2. Amount of hand sanitizer used by children aged ≤3 years at home or at school by product type as reported by the adult respondent.

Supplemental Table 3. Amount of hand sanitizer used by children aged ≥4 years at home or at school by product type as reported by the adult respondent.

Supplemental Table 4. Estimated systemic exposures (mg/kg-bw/day) pre- and during pandemic to unspecified chemical present in hand sanitizer at various concentrations and used by children aged 5 years. ^(1)^
